# Unique case of pelvic congestion syndrome caused by a new communication branch of the portal-vena cava system

**DOI:** 10.1186/s13019-023-02214-4

**Published:** 2023-04-10

**Authors:** Wei Zheng, Chun Sun, Jinming Yang, Yingfeng Wu

**Affiliations:** 1 Department of Vascular Surgery, Xuanwu Hospital, Capital Medical University, No. 45 Changchun Street, Xicheng District, 100053 Beijing, People’s Republic of China; 2 Department of Vascular Interventional, Aerospace Central Hospital, No. 15 Yuquan Road, Haidian District, 100049 Beijing, People’s Republic of China; 3 Department of Vascular Surgery, Luhe Hospital, Capital Medical University, No. 82 Xinhua South Road, Tongzhou District, 101199 Beijing, People’s Republic of China

**Keywords:** Congenital portosystemic shunt (CPS), Pelvic congestion syndrome (PCS), Vascular malformation

## Abstract

Congenital portosystemic shunt (CPS) is a developmental anomaly of the portal vein system. The disease can cause blood from the portal vein to flow into the vena cava, resulting in various atypical clinical manifestations. Pelvic congestion syndrome (PCS) caused by CPS is particularly rare. A young woman with PCS had an abnormal communicating branch of the left ovarian vein (OV). Her left OV drained normally into the left renal vein, and at the same time communicated with the portal vein, forming an extrahepatic portosystemic shunt. With embolization of her left OV, the patient was cured of PCS.

## Background

Chronic pelvic pain (CPP) often refers to intermittent or persistent pelvic discomfort that lasts for three to six months or longer [1]. The CPP of venous origin is called PCS. In the VEIN-TERM transatlantic interdisciplinary consensus document, PCS is defined as “chronic symptoms produced by ovarian or pelvic venous reflux or obstruction, including pelvic discomfort, perineal heaviness, urine urgency, and painful intercourse” [2]. A variety of mechanical and hormonal causes that result in venous dilatation (> 5 mm) and dysfunction may be involved in the varied etiology of PCS [3].

The OV, also known as the gonadal vein or reproductive vein, originates from the ovaries bilaterally, initially as a trailing plexus of veins that wraps around the ovarian artery and gradually merges into a single vein after leaving the pelvis. The bilateral OVs normally ascend next to the psoas major muscle. While the left OV initially enters the left renal vein at a straight angle and then feeds into the inferior vena cava, the right OV enters the inferior vena cava at an acute angle directly at the level of the right renal vein. Therefore high pressure in the left OV is not conducive to venous blood return and is prone to venous stasis, which is one of the most important causes of ovarian varicose veins [4]. Dilated OVs are seen in 10% of women, of whom up to 60% may develop PCS [2].

Pelvic vasculature is relatively complex and can exhibit variation among individuals. According to Gay et al., 40% of patients present multiple gonadal veins [5]. Variations of number of left side gonadal vein and their mode of termination are frequent [6]. According to a study done by Gupta et al., there were more variations of gonadal veins on left side with a male predominance [7]. Reports in the literature present limited knowledge concerning variations of the ovarian vein, particularly its duplication. Duplication and other variants of the ovarian vein are largely incidental findings during surgeries, anatomy course dissections, radiological procedures, and at autopsy [8].

CPS is a developmental malformation of the portal venous system that results in the flow of blood from the portal vein into the vena cava system. The malformation is thought to arise from the fourth to eighth week of embryonic development (the stage of development of the hepatic and somatic veins) and is therefore often associated with other cardiac and vascular malformations [9]. CPS are usually diagnosed in children but may be discovered at any age [10]. The overall prevalence of CPS is reported to be 1 in 30,000, and the rate of shunt vessels remaining open after infancy is 1 in 50,000. The age and trigger for being diagnosed with CPS varies from case to case [11].

In this paper, we report a case of a woman, whose left OV converges into the left renal vein while communicating with the portal vein, creating an extrahepatic portal shunt, which led to PCS.

## Case presentation

A 30-year-old unmarried and childless woman had recurrent lower abdominal cramping and pain for 4 years. This pain was worse after prolonged sitting and standing and would be relieved by a change in position. She thought that the pain was caused by dysmenorrhea and consulted a gynecologist. She had been treated with goserelin acetate, nerve block, and oxygen perfusion therapy, all to no avail. The patient then underwent a pelvic magnetic resonance scan, which showed tortuous intrapelvic vessels. She was diagnosed with PCS based on clinical history and the MRI findings. The patient came to our clinic for consultation. On examination, no abnormalities were found in the lower abdomen, perineum, and both lower extremities. Vascular ultrasonography showed no significant abnormalities in the deep veins of the lower extremities bilaterally.

**Fig. 1:**
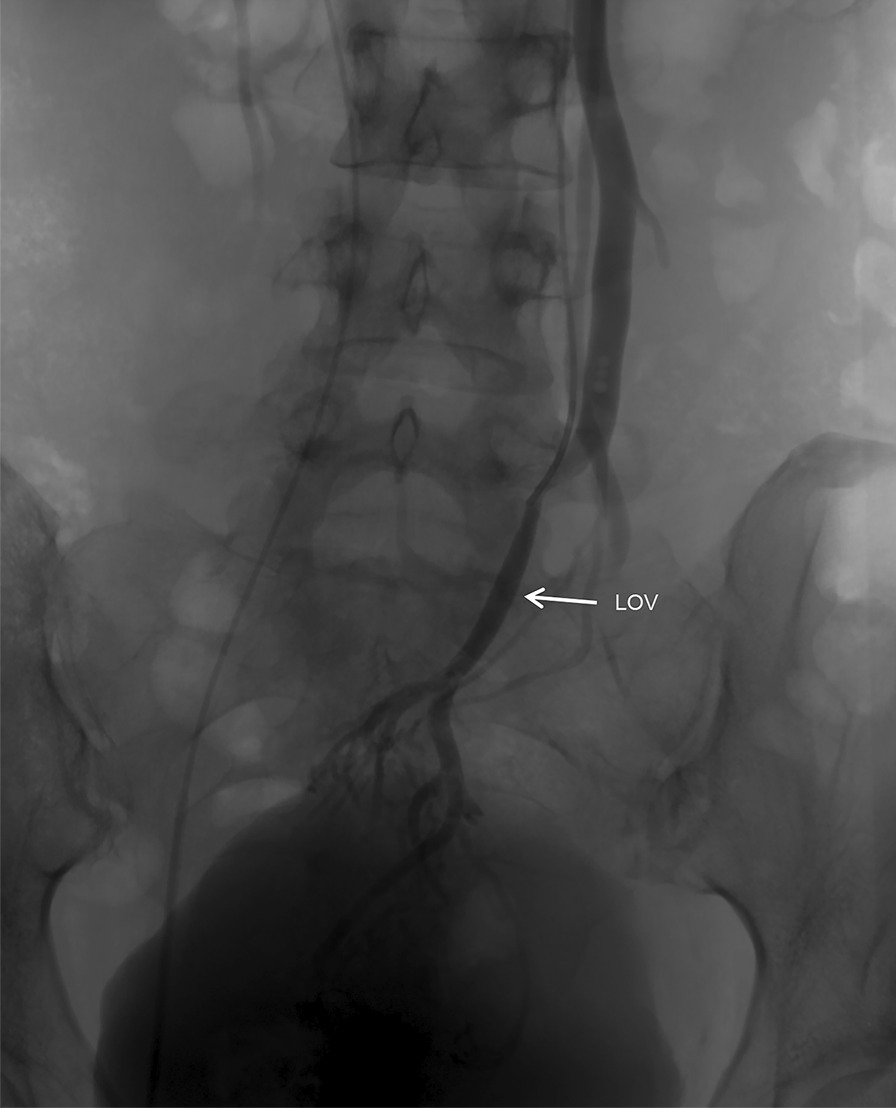
Venography showing the left ovarian vein is dilated, and the diameter of the thickest section is 7.5 mm

**Fig. 2:**
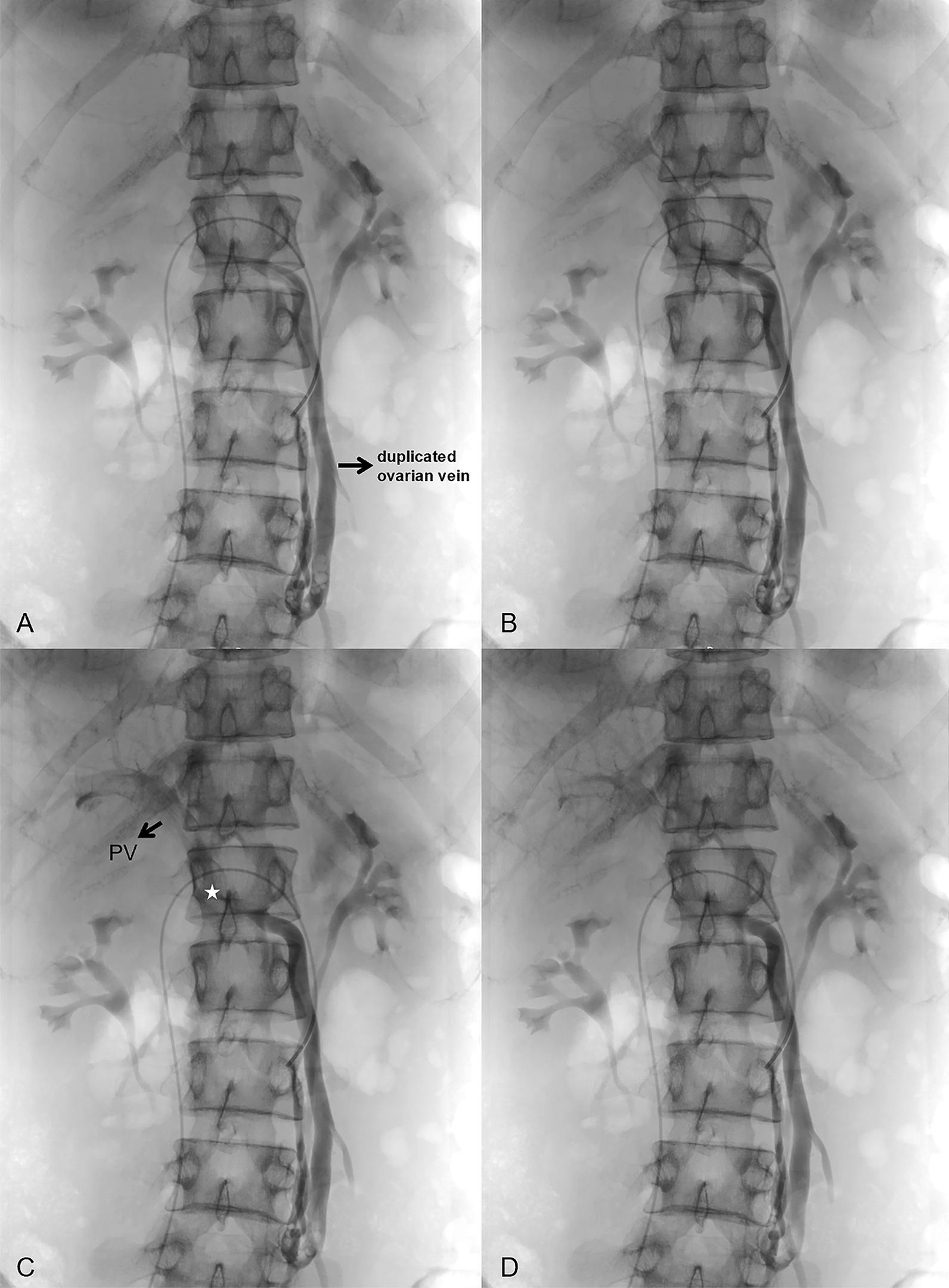
A front view showing a duplicated ovarian vein draining to the portal vein. The point of communication between the ovarian vein and the portal vein is marked with an asterisk (PV- portal vein)

**Fig. 3:**
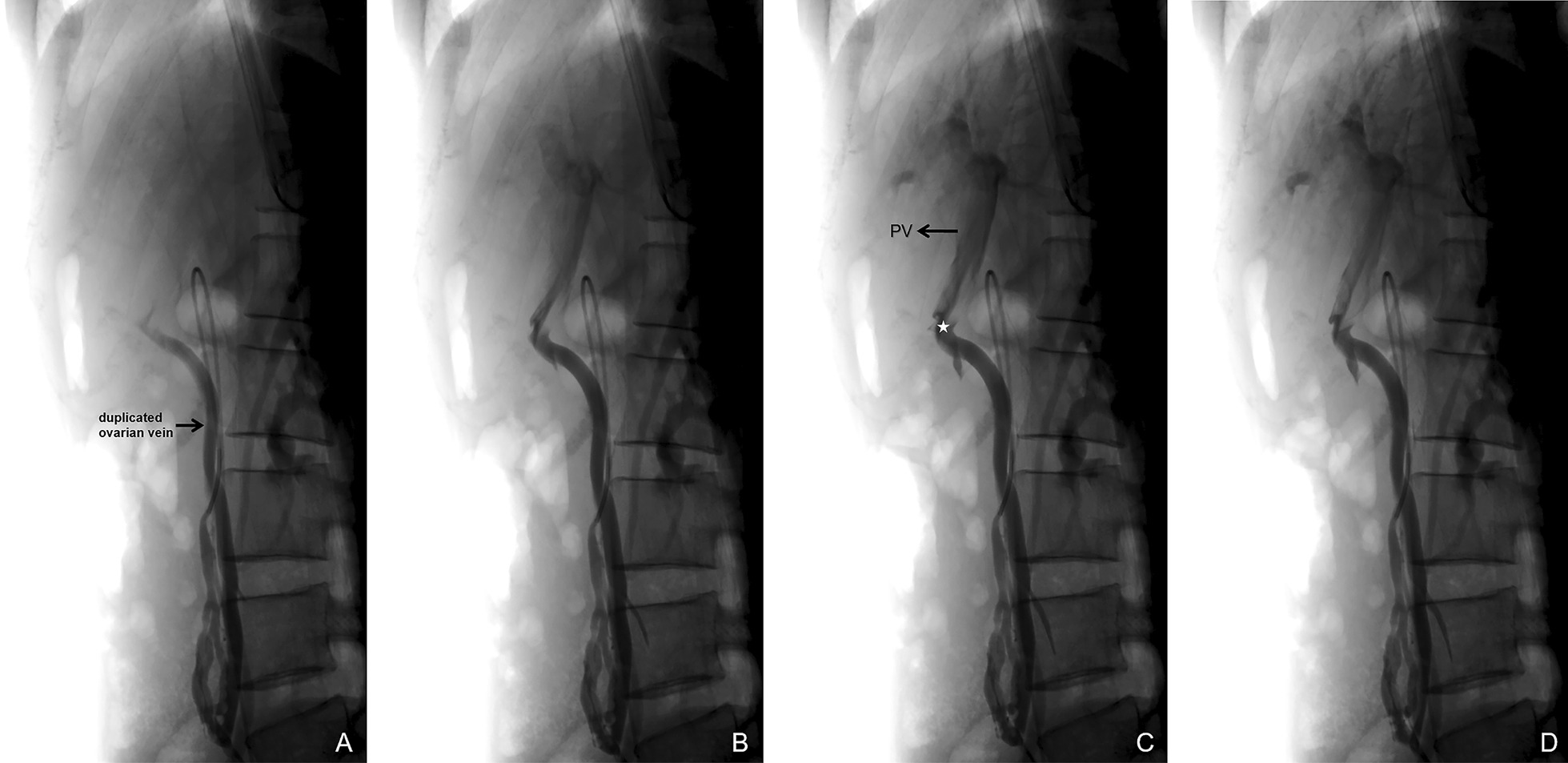
A side view showing a duplicated ovarian vein draining to the portal vein. The point of communication between the ovarian vein and the portal vein is marked with an asterisk (PV- portal vein)

**Fig. 4:**
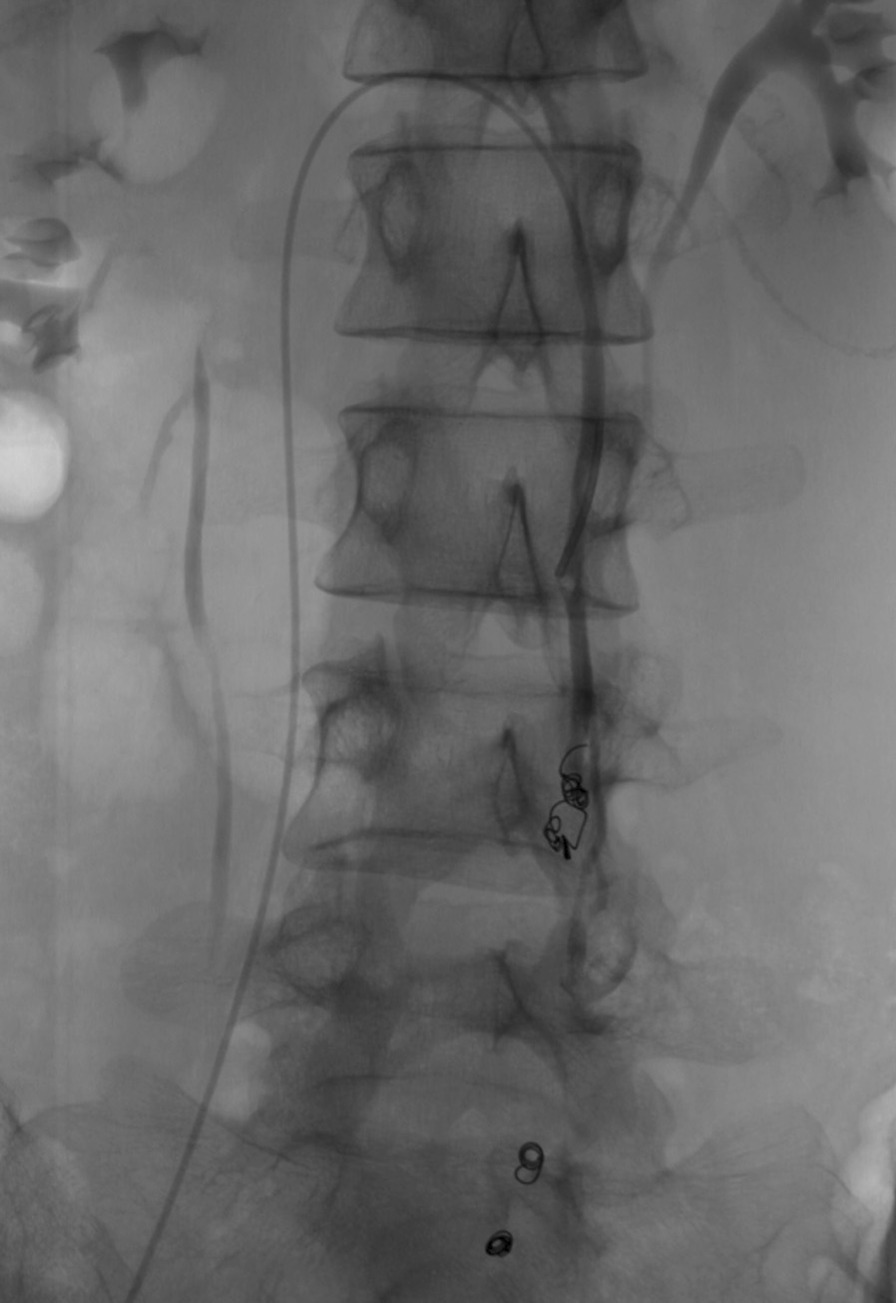
Venography image after left ovarian vein embolization

On venography, there was nothing abnormal detected in the iliac veins, lower limb veins, and renal veins. In the left OV, the contrast was seen to flow backward into the pelvis under the Valsalva maneuver, with localized venous tortuosity (Fig. 1). An anomalous traffic branch converging into the portal vein is seen in the lower part of the OV (Figs. 2 and 3). After obtaining consent, two COOK 18s-4/2 spring coils were placed in the intrapelvic portion of the distal OV. To block the reverse blood reflux from the ovarian and portal veins. No contrast reflux to the distal end of the embolus was seen on re-imaging, and embolization was satisfactory. To ensure definitive embolization, three COOK 18s-4/2 spring coils were placed in the trunk of the OV proximal to the confluence of the lateral branches of the portal vein. There is still a small lateral branch flowing back into the abnormal traffic branch (Fig. 4). Postoperative biochemical examination and ultrasonography of both kidneys showed no abnormality. The patient recovered well and was discharged 1 day postoperatively. Postoperative biochemical examination and ultrasonography of both kidneys showed no abnormality. The patient recovered well and was discharged 2 days postoperatively. The patient has been followed up for 18 months with no recurrence of preoperative symptoms.

## Discussion and conclusions

CPS is a rare malformation of the portal vein system. It was first described by Abernethy in 1793, so it is also called Abernethy malformation [6]. Morgan and Superina classified the disease into 2 types: type I (terminal-lateral shunt), in which portal venous blood flows completely into the vena cava; type II (lateral-lateral shunt ), in which portal venous blood is partially diverted into the liver and partially into the systemic venous system. The specific location of type II shunt vessels is varied, such as inferior mesenteric vein-inferior vena cava shunt, inferior mesenteric vein-iliac vein shunt, splenic vein-left renal vein shunt, etc. [7]. Hajime Takeuchi reported a case of Y-type portal shunt in which the inferior mesenteric vein, after dividing into the superior rectal vein then divided into 2 branches out of which one drained into inferior vena cava and other into the left OV [8]. Liao et al. reported a case of hemorrhagic shock in a patient. During dissection, they found the presence of a communicating branch of the splenic vein to the left OV. The rupture of this vessel caused a hemorrhage in the patient. They suggested that this traffic branch of the portal venous system could be considered the fifth branch of the portal-vena cava system [9].

In this case, we present a vascular malformation that is of subtype Abernethy Type II. This abnormality leads to the ovarian venous hypertension, producing blood reflux and tortuous dilatation of the veins, resulting in the patient’s symptoms of PCS. To our knowledge, this is the first report, suggesting that extrahepatic portal shunts may be associated with PCS. The pathophysiological and hemodynamic changes that occur are not known and require further study. Abernethy malformation can cause liver hypoperfusion and abnormal shunt of the extrahepatic venous system. This results in various atypical clinical manifestations, while the symptoms of severe damage to liver function are not obvious. Therefore, Abernethy malformation is not easily detected and diagnosed [10]. In our present case, the liver function was normal.

The use of transvascular interventional embolization in the treatment of type II Abernethy malformation has also been reported. Yamagami et al. performed embolization on a 6-year-old child with type II Abernethy malformation. The location of embolism was the inferior mesenteric vein- left internal iliac vein shunt. This treatment was clinically effective and minimally invasive [11]. In this case, by embolization of the left OV and the anomalous traffic branch, the portal shunt, and the left OV-left renal vein-inferior vena cava vascular pathways were blocked, ensuring that no reflux occurred in either pathway. The patient no longer showed symptoms of PCS after the operation, which proved the effectiveness of our embolization treatment.

A thorough understanding of vascular anatomical differences can aid doctors in correctly diagnosing and treating patients as well as comprehending the connection between disease and vascular malformation. In interventional embolization, understanding these variations can guide clinicians in preventing complications. Therefore, a good knowledge of vascular variants is important for clinical work.

In conclusion, we share a case of PCS due to an anomalous traffic branch of the left OV converging into the portal vein. And the patient was cured by embolization of the distal left OV and flow restriction of the OV-portal vein access. We hope that the report of a case with a specific variant of abdominal vascularity will increase the knowledge of the anatomy of this region and contribute to clinical management.

## Data Availability

All data analyzed during our study are included within the published article.

## List of abbreviations

CPS: Congenital portosystemic shunt
PCS: Pelvic congestion syndrome
CPP: Chronic pelvic pain
OV: ovarian vein
